# Carbonate framework and sediment production across island-fringing coral reef habitats and a natural nutrient gradient

**DOI:** 10.1038/s41598-026-49702-w

**Published:** 2026-04-24

**Authors:** Ines D. Lange, Marleen Stuhr, Chris T. Perry, Aitana Gea-Neuhaus

**Affiliations:** 1 Geography, Faculty of Environment, Science and Economy, University of Exeter, Exeter, UK; 2 Geoecology and Carbonate Sedimentology, Leibniz Centre for Tropical Marine Research (ZMT), Bremen, Germany; 3 Faculty of Science and Engineering, School of Biological and Marine Sciences, University of Plymouth, Plymouth, UK

**Keywords:** Coral reef carbonate budgets, Reef framework production, Sediment budgets, Sediment generation, Calcium carbonate production, Sediment composition, Reef island resilience, Seabird nutrient subsidies, Ecology, Ecology, Ocean sciences

## Abstract

Coral reef framework production and sediment generation are key controls on vertical reef accretion and reef island maintenance. However, quantitative data on these geo-ecological functions remain scarce, particularly within shallow habitats that mediate incoming wave energy and sediment supply to shorelines. This study quantified carbonate framework and sediment budgets across shallow lagoon reef (1–2 m), shallow forereef (2–3 m) and deep forereef habitats (8–9 m) surrounding six islands within three atolls (2 islands/atoll) in the remote Chagos Archipelago, Central Indian Ocean. One of the islands in each atoll supports dense seabird colonies that deliver natural nutrient subsidies to nearshore habitats via guano run-off, enabling assessment of nutrient impacts on reef carbonate dynamics. Benthic surveys (*ReefBudget* and *SedBudget*) were combined with sediment composition analyses from shallow reefs and adjacent beaches. Variation in coral cover and composition among atolls, linked to differences in atoll size and post-2016 bleaching recovery, resulted in higher framework production at Salomon Atoll. After accounting for atoll-level variability, framework production exhibited habitat-specific patterns, with highest mean rates at lagoon reefs (5.5 kg m^⁻2^ yr^⁻1^), followed by deep and shallow forereefs (3.5 and 3.1 kg m^⁻2^ yr^⁻1^, respectively). Reefs adjacent to seabird islands showed enhanced framework production at lagoon and shallow forereef sites (by + 3.7 and + 3.1 kg m^⁻2^ yr^⁻1^, respectively), driven by increased coral calcification and reduced parrotfish grazing. Sediment production was dominated by parrotfish erosion, with highest rates at deep forereefs (3.0 compared to 1.3 kg m^⁻2^ yr^⁻1^ at shallow reefs), little variation among atolls, and a limited response to nutrients. Sediment composition was dominated by reef framework constituents (coral and crustose coralline algae), with secondary contributions from *Halimeda*, molluscs and benthic foraminifera. Beach sediments resembled shallow reef sediments, both showing increased *Halimeda* near nutrient-enriched reefs due to past calcareous algal blooms. Our findings demonstrate how different spatial scales and seabird-derived nutrients modulate carbonate production in remote reef island systems.

**Supplementary Information:**

The online version contains supplementary material available at 10.1038/s41598-026-49702-w.

## Introduction

The production and erosion of calcium carbonate (CaCO_3_) is one of four important ecologically mediated functions that operate in coral reef ecosystems^1^. While physical and chemical processes also influence carbonate cycling, biological processes underpin the construction of the physical framework structure of reefs (on which all other taxa depend) and sustain the generation of biogenic carbonate sediments (which typically dominate nearby lagoons, beaches and reef islands). Reef framework production is driven predominantly by corals but is augmented by inputs from other calcareous taxa such as crustose coralline algae (CCA)^2,3^. At the same time, various species of fish, sea urchins and endolithic (internal substrate dwelling) taxa erode the carbonate framework through their feeding activities^4^. The net balance between these biological production and erosional processes is defined as a reef’s carbonate budget^2,5^ and strongly influences vertical reef accretion potential^6^. Many of the eroding processes also convert reef framework carbonate to sediment, which is excreted by bioeroding species of fish, urchins and endolithic sponges^7–9^. This sedimentary carbonate then contributes, along with the skeletal remains of shelly fauna (e.g., molluscs, foraminifera) and carbonate secreting algae (e.g., *Halimeda*) to the pool of biogenic sediment generated on and around reefs^10^.

Two distinct but linked components of the biological reef carbonate production and cycling system can thus be discerned, the primary framework producing component (the reef framework budget) and the biogenic sediment component (the reef sediment budget). Whilst significant research has been directed at quantifying reef framework budgets (reviewed in^11,12^), including recent efforts to quantify responses to long-term environmental change^6,13^, and the impacts of severe thermal stress events^14,15^, data on reef sediment budgets are relatively sparse. There are even fewer examples of studies seeking to quantify both framework and sediment budget components on the same reefs (but see^16–20^). This combined framework and sediment budget approach is relevant because the process of vertical reef accretion is driven not only by framework production but also by sediment inputs, which can comprise more than 50% of the internal structure of reefs^21^. Both components of reef carbonate production are also important to assess when considering the larger spatial connection between reefs and associated landforms^12,22,23^. Reef framework structures can modulate across-reef wave energy transfer and mitigate coastal flooding^24–26^, and sediment supply potential is important for sustaining proximal shorelines and reef islands^27–29^. Shallow reef crest habitats are especially critical in this regard, but data on framework and sediment production rates in these reef areas is scarce^11^. Improving our understanding of the magnitudes of these two carbonate budget components, how rates vary across different spatial scales and environmental gradients, and how rates may be responding to future climate change driven perturbations is thus an important area for focus.

Here, we quantify both carbonate budget components across a range of reef habitats and a natural nutrient gradient in the remote Chagos Archipelago, Central Indian Ocean. Specifically, we explore framework and sediment carbonate production rates on shallow lagoon reefs (1–2 m depth), shallow forereefs (2–3 m depth) and deep forereefs (8–9 m depth) surrounding islands with and without dense seabird colonies. The presence of seabirds on islands strongly enhances the nutrient supply to surrounding reefs through guano run-off^30,31^, which has been shown to boost coral growth and calcification^32,33^ as well as parrotfish growth and biomass^34^. With this study we aim to address the paucity of data on integrated framework and sediment budgets in a location largely free from direct anthropogenic disturbance, and to explore whether seabird-derived nutrient enrichment influences rates of carbonate production.

## Material and methods

### Study site

**Fig. 1:**
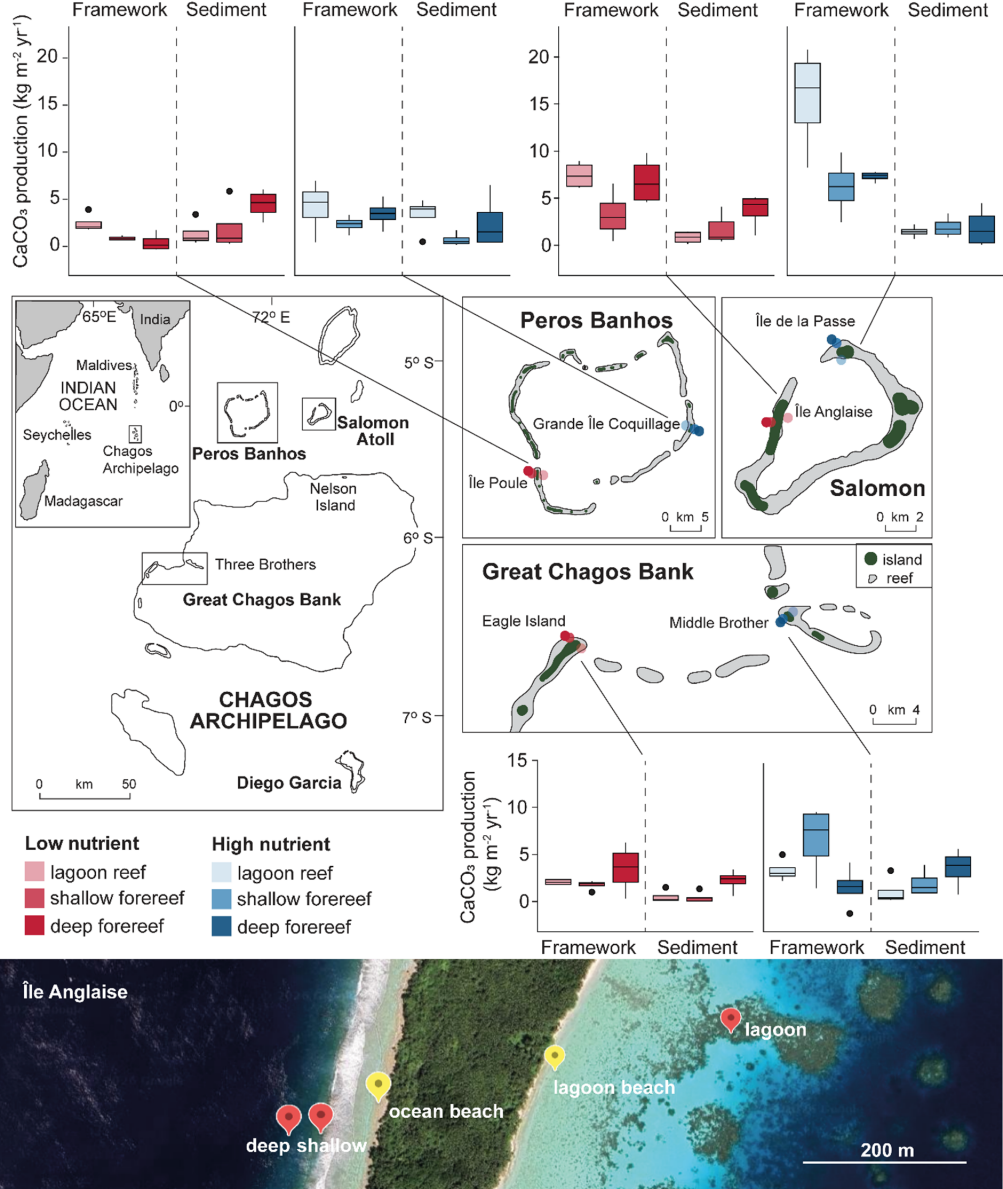
Site-level reef framework budgets and reef sediment budgets. Comparison of carbonate framework production (Framework, in kg CaCO_3_ m^-2^ yr^-1^) and carbonate sediment production (Sediment, in kg CaCO_3_ m^-2^ yr^-1^) across atolls (Salomon, Peros Banhos, Great Chagos Bank), habitats (lagoon reef, shallow forereef, deep forereef) and nutrient status (low, high; linked to seabird densities on islands). Boxplots display median (horizontal line), first and third quantiles (box edges), range of values no further than 1.5*inter-quantile range (whiskers) and outliers (points) at each site (*n* = 4 transects). Panel at the bottom depicts sampling design across islands using Ile Anglaise as an example (image from GoogleEarth version 10.104.67.1, Location: 5° 19′ 43.11″ S 72° 13′ 18.14″ E, data from Airbus imagery 22/10/2025, retrieved from https://earth.google.com/web/ on 11/03/2026).

Our study was conducted in the remote Chagos Archipelago, Central Indian Ocean. This large marine protected area experiences minimal direct anthropogenic influence and provides the opportunity to study reef functioning across spatial and environmental gradients largely free of external stressors^35^. Surveys were conducted in 2023 around six low-lying reef islands within three atolls (2 islands per atoll, Fig. 1). The atolls vary in size (Great Chagos Bank > Peros Banhos > Salomon), but all are influenced by seasonal trade winds, with peak wind speeds and wave exposure occurring during the southeast monsoon. Coral reefs across the entire Archipelago were severely affected by the 2015–2016 bleaching event, with coral cover on fore reefs decreasing from > 40% to < 10%^36^. Reefs in Salomon atoll lagoon suffered less severe coral mortality (6% reduction compared to 42% at Great Chagos Bank and 72% at Peros Banhos), likely due to heat adaptation in the relatively small, enclosed lagoon^37^. Fore reefs recovered to ~ 20% absolute coral cover in 2021^14^ and 30–50% coral cover in 2023 (this study), indicating that reefs have approximately returned to pre-bleaching conditions. To assess the influence of natural nutrient subsidies on geo-ecological reef functions, we compared one island with high seabird densities (20–4000 breeding pairs ha^-1^), and one island with low seabird densities (< 1 breeding pair ha^-1^) in each of the three atolls (details on island size and seabird densities in^31^). These seabird islands are characterized by ~ 250 times higher nitrogen inputs compared to islands with few birds, which subsequently flow to near-shore reefs and are taken up by coral reef organisms^30^. Previous work has shown that these seabird nutrients increase fish growth and biomass^34^ as well as rates of coral growth and calcification on surrounding coral reefs^32,33^. At each island, we surveyed carbonate framework and sediment production at shallow lagoon reefs (1–2 m depth), shallow forereefs (2–3 m depth) and deep forereefs (8–9 m depth), totalling 18 survey sites (Fig. 1). Sediment was sampled at each survey location and adjacent beaches.

### Reef framework budgets

Carbonate framework production was assessed using the Indo-Pacific *ReefBudget* methodology^38^ (available at http://geography.exeter.ac.uk/reefbudget/). For benthic transects (*n* = 4/survey site), coral colonies (identified to genus and morphological level) and other substrates (crustose coralline algae (CCA), turf algae, fleshy macroalgae, sediment, rubble, sponges and other benthic organisms) were measured to the nearest cm following the reef contour directly beneath a 10 m guide transect line by using a separate flexible tape. Survey data was entered into the *ReefBudget* spreadsheets, which calculate benthic community composition (% cover) and combine the morphology and size of individual coral colonies with genera/morphotype-specific calcification rates to estimate annual coral carbonate production (Coral G with G = kg CaCO_3_ m^-2^ yr^-1^). Locally collected linear growth rate and skeletal density data^14^ were used for dominant taxa. As increased coral growth and calcification rates in response to seabird nutrients were found at the study sites^33^, we adjusted Coral G at lagoon and shallow forereef sites close to the seabird islands to reflect enhanced carbonate production close to shore, while deeper forereef sites were considered unaffected due to the dilution of nutrients further off shore^39^. Carbonate production at shallow seabird sites was multiplied by 2.7 for fast-growing (competitive or weedy) coral taxa and by 1.6 for slower growing (generalist or stress-tolerant) taxa (differences in calcification rates for *Acropora vermiculata* and *Isopora palifera*, respectively^33^) before being summed to Coral G. Although these calcification rates were derived from Salomon atoll only, *Acropora* translocation experiments showed consistent growth responses to seabird nutrients across the entire Archipelago^32^, justifying the extrapolation of calcification rates to other atolls. Cover of CCA was multiplied with locally determined calcification rates (0.042 ± 0.004 g cm^-2^ yr^-1^ at seabird islands, 0.070 ± 0.006 g cm^-2^ yr^-1^ at islands without seabirds)^38^ to estimate CCA carbonate production (CCA G). CCA calcification rates were quantified by dissolving carbonate crusts accreted on PVC tiles with 10%HCl after one year of exposure at Salomon lagoon sites (following^38^). CCA G was added to Coral G to yield Gross Production G.

Sea urchin abundance and size (to nearest cm) were surveyed in 2 m belt transects along the same transect lines and combined with published taxa- and size-specific erosion rates to calculate urchin carbonate erosion (Urchin G). Parrotfish, pufferfish and triggerfish abundance and size (to nearest cm) was quantified along four separate belt transects (30 × 5 m) and parrotfish erosion was estimated using local feeding metrics for most species^40^. Decreased bite rates at seabird islands compared to islands without seabirds were observed for two dominant parrotfish species (0.8-times lower, Rodriguez-Fillol et al. in preparation), and thus parrotfish erosion at lagoon and shallow seabird sites was multiplied by 0.8. Including these site-specific bite rates decreases parrotfish bioerosion and sediment production estimates by 20% at lagoon and shallow forereef sites close to seabird islands (0.44 and 0.27 kg m^-2^ yr^-1^, respectively). While this lowers the likelihood of a positive seabird nutrient effect on sediment production in our analyses, we prefer to use site-specific rates for more accurate and conservative results. Erosion by each functional group was summed at transect-level, except fish erosion which was determined along separate transects and was therefore factored in as an average value, to yield Gross Erosion G, which was subtracted from Gross Production G to yield the net carbonate budget (Net G).

### Reef sediment budgets

Carbonate sediment production was estimated using the *SedBudget* methodology^10,41^ (available at http://geography.exeter.ac.uk/sedbudget/), with benthic surveys modified from handbook recommendations (using a quadrat) to integrate better with *ReefBudget* surveys (using a transect line). Specifically, gastropods, bivalves and calcifying algae were counted and measured in belt transects of 1 m each side of the same transect line used for *ReefBudget* surveys (or 0.5 m if very high densities of calcifying algae were present) during a separate pass over the transect. Abundances and sizes were combined with published taxa-specific carbonate content and turnover rates to yield sediment production rates for each taxonomic group. Additionally, taxa-level data on the contribution of produced sediments to different grain size classes were used to predict grain size distributions of sediment production estimates. Benthic foraminifera abundance in reef surface sediment samples was quantified for each grain size fraction ≥ 125 µm (see *Sediment composition*) and normalized by the number of grains analysed in each fraction, thereby correcting for unequal counting effort. Normalized foraminifera abundances were multiplied by a productivity conversion factor for reef and rubble habitats^42^ to yield carbonate production rates.

Sediment production by sea urchins and parrotfish equals respective erosion rates, assuming that none of the carbonate is dissolved in the gut^8,43^, and grain size contributions for these taxa were estimated using the relevant species-specific proportions in the *SedBudget* calculation sheets. Additionally, pufferfish and triggerfish sediment production were estimated using published rates, but contributions were very small compared to parrotfish as abundances were low. Sediment produced by parrotfish, sea urchins and sponges were translated to estimates of coral (parrotfish: 95%, urchins: 55%, sponges: 100%) and CCA constituents (parrotfish: 5%, urchins 45%) using published data on proportions of grain types in faecal samples from the Indian Ocean (following^10^).

Sediment dissolution is likely minimal and may even be offset by post-depositional carbonate precipitation as observed in coral dominated habitats^19^. Our analysis therefore focused on the production side of the sediment budget, which is then available for transport through the system and/or for deposition within various sediment sinks (e.g., lagoons, beaches)^12^.

### Sediment composition and grain size distribution

Sediment samples (~ 200 g) were collected from survey locations (shallow forereef: 3–5 m, deep forereef: 8–10 m), as well as from upper beach deposits close to both lagoon and forereef sites in 2021 (Fig. 1), as part of a larger sediment sampling effort across the Archipelago. As the depth of lagoon sites sampled in 2021 (0.5 and 3–5 m) did not match with survey sites in 2023, we collected and analysed additional sediments from these sites in 2023 (lagoon: 1–2 m). Sediment accumulated on reef surfaces represents an integrated signal over several years of production, and sediment composition was found to be stable over short-to-medium timeframes (months to a few years), even following a cyclone^44^. Due to the absence of major stressors between 2021 and 2023, the comparison of samples and survey data is thus unlikely to be biased by short-term variability. Sediments were rinsed in freshwater and dried for subsequent laboratory analyses. Lagoon samples taken in 2023 were rinsed over a 63-µm sieve following *SedBudget* recommendations and are thus missing < 63 µm size classes. Since these fractions have been found to be very minor in forereef samples (< 1%, this study) and in a previous analyses of lagoon samples from a subset of sites^10^, impacts on subsequent analyses are considered minor. Dried samples were divided using a riffle splitter and a consistent mass of the working half was dry-sieved into the following grain size fractions following the Udden-Wentworth Scheme^45^ (> 2000 μm (pebble, gravel), 1000–2000 μm (very coarse sand), 500–1000 μm (coarse sand), 250–500 μm (medium sand), 125–250 μm (fine sand), 63–125 μm (very fine sand), < 63 μm (silt and clay)). Sediment size fractions were weighed, and grain-size distributions were used to calculate mean grain size and sorting (φ, geometric standard deviation (SD))^46^, where φ = –log₂(diameter in µm). Sorting was also calculated for *SedBudget* estimated grain size distributions, using only size classes > 63 μm to allow for a direct comparison to reef and beach sediments.

Grain size fractions finer than 250 µm (on average 15.8% across all samples; ~ 19% in beach and ~ 13% in reef sediments) are often difficult to identify reliably under the binocular microscope and were therefore not included in the general compositional analysis. The 125–250 µm fraction was, however, included for the benthic foraminifera analysis (see *Reef sediment budgets*), as foraminiferal tests are abundant and easily identifiable in this size range. For each sample, all available or at least 200 grains per grain-size fraction were picked and visually identified under a binocular microscope (Leica S6 E). Sediment particles were classified into the following categories^47^: coral, CCA, molluscs, crustacea, echinoids (urchin and seastar fragments), sponge/octocoral spicules, foraminifera, *Halimeda*, bryozoa, serpulids, ostracods, organoclasts, plastic, and unidentified bioclasts. Contributions by bryozoa, serpulids, and ostracods were very minor and grouped into “Other” with unidentified carbonate bioclasts. Plastic and organoclasts (vegetative plant remains) were removed from the analysis and total fraction of other components recalculated to focus on biological carbonate composition. Relative abundances were expressed as the percentage of each component within its respective grain-size fraction, and mean values were calculated across fractions weighted by the proportional mass of that fraction in the total sediment to describe the overall sediment composition.

### Statistical analysis

The effects of habitat (lagoon reef, shallow forereef, deep forereef) and nutrient status (low nutrient/seabird density, high nutrient/seabird density) on carbonate framework production and sediment production were investigated in a Bayesian framework with atoll (Salomon, Peros Banhos, Great Chagos Bank) as random factor and island nested within atoll (framework/sediment production ~ habitat * nutrient status (1|atoll/island)). Bayesian models can perform better than frequentist methods with small sample sizes but can be sensitive to prior specifications^48^. To be conservative, we used the recommended half-Cauchy variance prior and default (weakly uninformative) priors for both habitat and nutrient status (as not much prior data exists on their effects on carbonate production). All models were run for four chains, with 3,000 iterations and 1,000 warm-up iterations per chain, and checked for model convergence and fit using posterior predictive checks, traceplots, and the Gelman-Ruban convergence diagnostic (R-hat)^49^. All analyses were conducted in R 4.3.1^50^ and Bayesian models were implemented in STAN using the *brms* package^51,52^, along with the *tidybayes*^53^ and *emmeans*^54^ packages.

Differences in benthic community composition and sediment composition were explored via principal component analysis (PCA) using packages *FactoMineR*^55^ and *factoextra*^56^ and differences between groups were tested via PERMANOVA in packages *vegan* and *pairwiseAdonis*^57^.

## Results

### Reef framework budgets

**Fig. 2:**
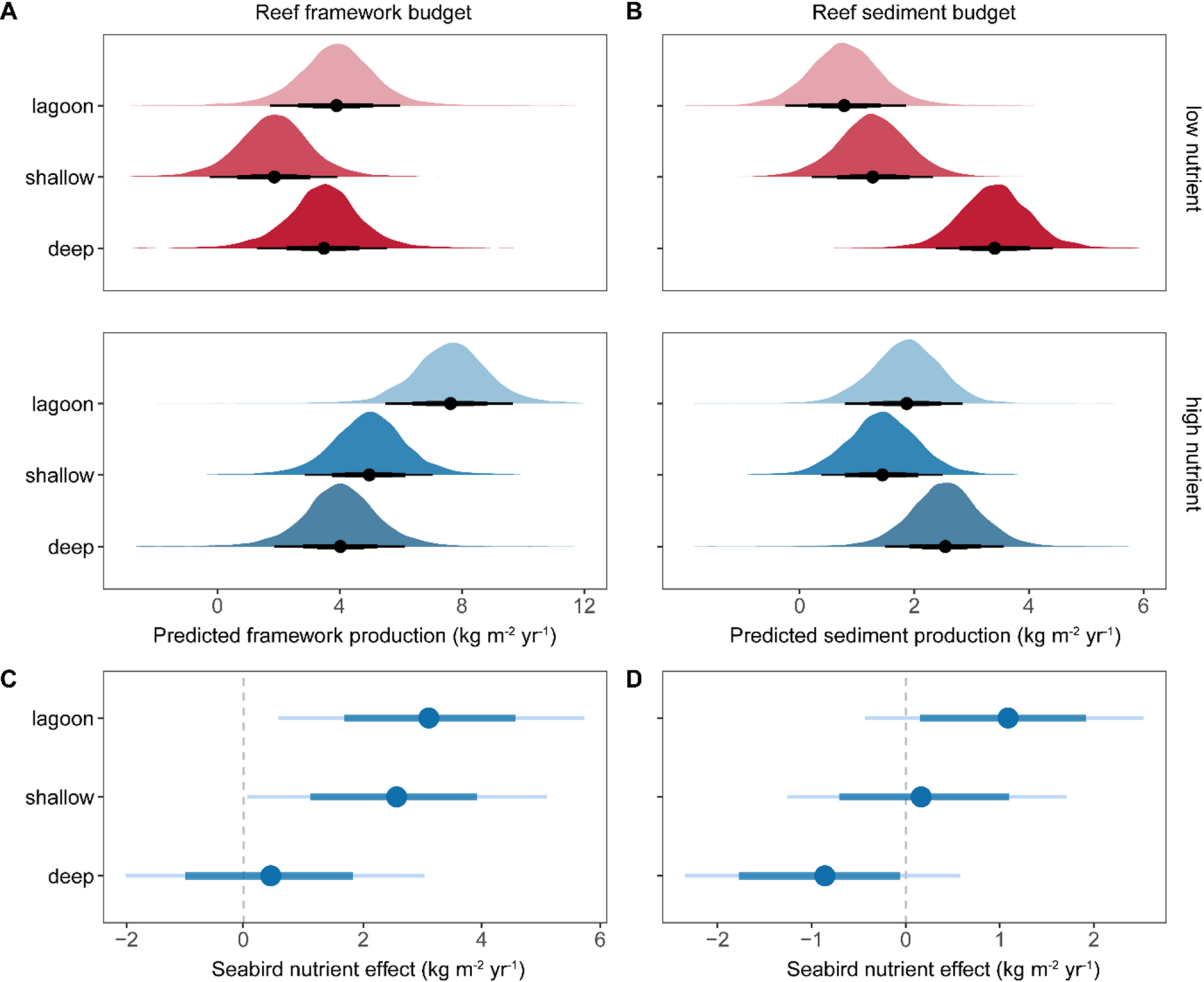
Effects of reef habitat and nutrient status on carbonate production. Posterior distributions for (**A**) reef framework budgets and (**B**) reef sediment budgets, comparing habitats (lagoon reef, shallow forereef, deep forereef) at low nutrient (red) and high nutrient (blue) sites (linked to seabird densities on islands). Conditional effects of seabird nutrient input on (**C**) reef framework budgets and (**D**) reef sediment budgets, with points right of the dashed line indicating a positive effect of seabird nutrients on carbonate production. Points represent median estimates, and lines represent 90% and 70% highest posterior density intervals (HPDIs).

Total coral cover and benthic community composition differed across atolls and habitats, but not according to seabird nutrient status (Supplementary Figs. S1 and S2). Due to the dominance of fast growing *Acropora* spp. at Salomon atoll (up to 40% total cover), carbonate framework production was higher compared to other atolls (Fig. 1), despite similar overall coral cover at deep forereefs in Peros Banhos and the Great Chagos Bank (Supplementary Fig. S1). After accounting for the variability at atoll level, lagoon sites showed higher reef framework budgets compared to forereef habitats (median estimates averaged over nutrient status [95% HPDI]: lagoo* n* = 5.49 [1.88–8.92], shallow = 3.14 [− 0.46–6.65], deep = 3.49 [− 0.10–6.97] kg m^-2^ yr^-1^), although this effect was more pronounced at high nutrient sites (Fig. 2A). Both lagoon and shallow forereef sites showed strong evidence for a positive seabird nutrient effect (Posterior probability (PP) = 0.97 and 0.95), with 3.73 [0.67–6.86] and 3.11 [0.12–6.20] kg m^-2^ yr^-1^ more framework carbonate produced close to seabird islands (Fig. 2C). Deep forereef sites only showed weak evidence for a positive seabird nutrient effect (PP = 0.64).

### Reef sediment budgets

Carbonate sediment production was similar across atolls (Fig. 1), but differed across habitats, with highest rates at deep forereef sites (median estimates averaged over nutrient status [95% HPDI]: lagoon = 1.29 [− 0.07–2.73], shallow = 1.34 [− 0.16–2.64], deep = 2.95 [1.60–4.36] kg m^-2^ yr^-1^). Differences between habitats were more pronounced at low seabird nutrient sites (Fig. 2B), where parrotfish biomass at deep forereefs was especially high (Supplementary Fig. S1). A moderate positive nutrient effect was evident for the lagoon sites (PP = 0.89), which on average produced 1.09 [− 0.86–2.89] kg m^-2^ yr^-1^ more sediment if located close to a seabird island, while shallow forereefs only showed weak evidence for a positive effect (PP = 0.58) and deep forereefs showed moderate evidence for a negative seabird nutrient effect (PP = 0.85) (Fig. 2D).

### Sediment composition

**Fig. 3:**
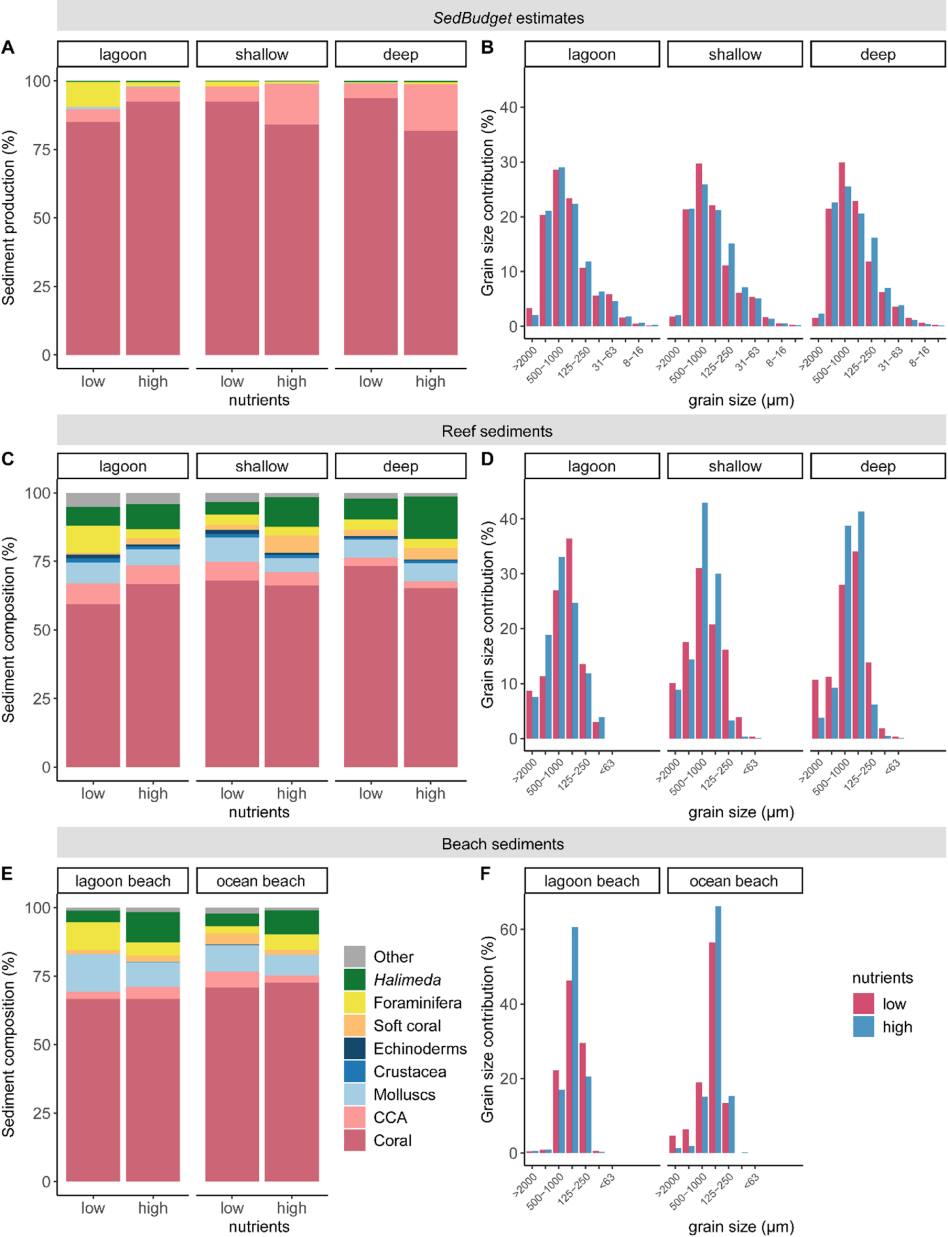
Sediment composition and grain size distribution. Composition of (**A**) estimated sediment production calculated from *SedBudget* surveys, (**C**) sediment sampled at the same reef locations, and (**E**) sediment collected from upper beaches close to survey locations (**C** and **E** for grain size classes > 250 µm). Grain size distributions for (**B**) estimated sediment production, (**D**) on-reef sediments, and (**F**) upper beach sediments. Plots compare compositions and distributions across reef habitats (lagoon reef, shallow forereef, deep forereef) or beach habitats (lagoon beach, open ocean-facing beach) and nutrient status (low, high; linked to seabird densities on islands).

Reef sediment budgets identified parrotfish and sea urchins as the main sediment producers. As these organisms convert primary reef framework carbonate to sediment through bioerosion, sediment composition estimated from *SedBudget* surveys was strongly dominated by coral and CCA fragments at all sites (mean ± SE: 97 ± 1%, range: 86–99%,). CCA abundance was especially high at sites where we recorded large *Diadema* sea urchins (e.g., forereefs of Middle Brother, a high nutrient location) (Fig. 3A). Although also dominated by coral and CCA, around 30% of constituents in on-reef sediment samples collected from the same sites were contributed, in decreasing order, by *Halimeda,* molluscs, benthic foraminifera, soft corals, crustaceans and echinoderms (Fig. 3C). High nutrient sites on average showed higher relative contributions by *Halimeda* (high: 9–15%, low: 4–8%) and soft coral spicules (high: 2–6%, low: 1–2%), although overall sediment composition was not significantly different (Supplementary Fig. S3). CCA fragments were generally more abundant at shallow lagoon and forereef habitats (5–8%) compared to deep forereefs (2–3%). The composition of beach sediments was very similar to reef sediments (Fig. 3E) and also characterized by elevated abundances of *Halimeda* constituents at high nutrient sites for both lagoon and open ocean-facing beaches (high: 9–11%, low: 4–5%). Mollusc fragments were slightly more abundant in beach samples (10 ± 2%, range: 4–28%) compared to reef sediments (7 ± 1%, range: 4–13%). Overall, reef carbonate framework components (i.e., coral and CCA) remained the dominant source of sediment constituents on beaches (72 ± 4%, range: 46–88%).

### Grain size distribution

Sediment production estimates predict grain sizes down to < 8 µm, resulting in relatively small mean grain sizes (402 ± 75 µm). Containing very little fine material, reef sediments were characterized by overall higher mean grain size (635 ± 183 µm) and larger contributions of gravel (> 2000 µm), while beach sediments were dominated by medium sand (418 ± 150 µm) (Fig. 3B,D,F). Beach sediments were much better sorted (moderately well, φ SD = 0.66 ± 0.15) than reef sediments (moderate to poor, φ SD = 1.07 ± 0.21), indicating substantial sorting during the transport from reefs to beaches, or on the shoreline itself. Sorting was similar across habitats and nutrient status for both sediment production estimates and sediment composition on reefs and beaches (Supplementary Fig. S4).

## Discussion

Understanding rates of both carbonate framework and sediment production is essential for assessing prevailing states of reef building and shoreline sediment supply. Predictions of reef carbonate cycling regimes have increasing relevance in the near future, when ecological reef degradation is expected to shift benthic community compositions and reduce carbonate production^12^. Our results show variability in both reef framework and reef sediment budgets across different spatial scales (atolls, islands, and habitats around islands) and demonstrate how natural nutrient subsidies can boost reef geo-ecological functions. Framework production exceeded sediment production at sites with high cover of branching corals but were of similar magnitude at other sites, emphasizing the importance of both processes in the context of overall reef carbonate cycling regimes.

Spatial and temporal context plays a crucial role in regulating carbonate production. The overall high coral cover and dominance of fast-growing *Acropora* spp. at Salomon atoll reflected less severe coral mortality during the 2015–2016 bleaching event (at lagoon reefs^37^), and rapid recovery afterwards (at forereefs^14^), increasing carbonate framework production compared to other atolls across all habitats. Habitat effects varied across studied islands, likely reflecting interactions between nutrient availability and other environmental factors that were not explicitly accounted for, such as atoll size, wave exposure and reef structure. However, strong seabird nutrient effects on framework production rates were evident at both lagoon and shallow forereefs despite similar coral cover and benthic community composition at paired sites within atolls. These differences were almost exclusively driven by elevated coral calcification rates at high-nutrient locations, which is consistent with previous work demonstrating enhanced process variables (e.g., coral growth and calcification, recovery speed after disturbance) around seabird-rich islands without corresponding differences in status variables (i.e. coral cover)^32,33^. Despite lower parrotfish feeding rates around seabird-rich islands (likely due to higher nutrient density in feeding resources, Rodriguez Fillol et al. in prep.), a positive seabird nutrient effect was also evident for sediment budgets at lagoon reefs due to higher parrotfish biomass. Elevated parrotfish biomass around seabird-rich islands has been shown in previous studies^30^. Because bioerosion rates strongly scale with fish size^58^, the effect on sediment production is likely further amplified by ~ 35% faster parrotfish growth rates around seabird islands^34^. While supporting findings from these previous studies that observed positive seabird nutrient effects on reef ecology, our observations also highlight the subtle but important role of localised natural nutrient subsidies in shaping geo-ecological functions of near-shore coral reefs. Although not calculated here, it is well established that coral cover, community composition, calcification rates and sediment production all strongly influence vertical reef-building potential^6^. Understanding how reef accretion rates respond to spatially variable environmental conditions has broader implications for evaluating the interactions between reef ecology, reef framework development, and wave energy transformation, all of which underpin the coastal protection benefits offered by many reef systems^26^.

Nearshore wave processes as well as ecologically mediated sediment supply control reef island persistence and growth^59^. Unlike in near-continental or high atoll island settings, where terrigenous sediment inputs can be important, almost all sediment accumulating on low-lying reef islands is biogenic in origin and sourced from the adjacent reefs^23,60^. Biological sediment production rates similar to our study were found around Maldivian reef islands (3.5–5.2 kg m^-2^ yr^-1^ on reef patches, 0.1–1.8 kg m^-2^ yr^-1^ on nearshore sites and rubble ridge zone)^18^. The dominance of coral fragments and crustose coralline algae (CCA) constituents in sediments is also consistent with previous regional estimates^22,28,59^, and reflects the importance of parrotfish and bioeroding urchins that drive reef framework breakdown. While parrotfish almost exclusively produce coral sand^43^, sea urchins commonly graze on CCA covered substrate^61,62^, explaining higher estimates of CCA fragment abundance at sites where we recorded large *Diadema* sea urchins. In contrast to sediment production estimates, reef and beach sediments represent an integrated signal over space and time, shaped by preferential transport of lower density or more hydrodynamically buoyant grains such as *Halimeda* segments and foraminiferal tests^63,64^. These transport processes can partially explain the higher diversity of constituents in sediment samples. Another contributing factor is likely the underestimation of sediment production by molluscs and benthic foraminifera in *SedBudget* calculations, which occurs due to the challenge of accurately quantifying small cryptic species in visual surveys and the paucity of data on site-specific productivity rates^10,65^. The elevated abundance of *Halimeda* segments in reef sediments at high nutrient sites most probably reflects legacy inputs from a short period of very high *Halimeda* cover next to seabird islands following the 2015–2016 bleaching event (40–50% cover^37^), even though present-day densities are low (mean cover: 2 ± 1%, range: 0–8%). Similar increases in *Halimeda* cover and sediment generation post-bleaching were observed in the Maldives^66^, illustrating how past ecological states can leave lasting imprints on reef sediment composition and connectivity between reef and island systems. This decoupling between present-day benthic cover and island sediment composition is consistent with findings from the Spermonde Archipelago, where *Halimeda*-rich sediments preserved in island cores reflect long-term reef ecological trajectories rather than contemporary *Halimeda* abundance alone^67^.

Fluxes of sediment delivery to reef-associated beaches depend not only on sediment production rates, but also on sediment sources and inherent grain size generation, because grain shapes and sizes control transport and deposition dynamics^64^. Sediments excreted by parrotfish make up most of the fine material in the sediment production estimates, and much of this fine sediment is dispersed when parrotfish excrete in the water column (20% loss^8^) or off the reef slope (11% loss^68^). Even if settling on the reef surface, finer material can get mixed into deeper sediment layers or accumulate within lower energy intra-framework cavities^69^, which together with the potential loss of the finest size fractions during sampling may explain the small contributions by fine sediments in reef sediment samples compared to census-based estimates of sediment production. The higher proportion of coarse gravel-sized material in reef sediments likely results from physical coral breakage, a process not presently captured in *SedBudget* estimates. Despite these discrepancies we found an overall strong correspondence between the types and grain sizes of sediment generated on the reefs and those actively contributing to island beaches. Beach sediments were better sorted than reefal deposits, consistent with patterns observed in the Maldives, where island beaches are composed predominantly of fine, well-sorted coral sand, with lesser contributions from CCA, abraded *Halimeda* fragments, and molluscan material^22^. Comparable reef-to-island sediment connectivity and hydrodynamic filtering have also been documented across the wider Indo-Pacific, where island beach deposits represent a selectively transported subset of reef-derived sediments^70–72^.

### Outlook

This study presents carbonate framework and sediment production rates from the shallow reef habitats most influential in mediating wave energy attenuation and sediment generation, thereby linking reef function to island development and resilience to future change. Of most concern in this context is that coral bleaching events associated with climate change have already started to reduce the abundance of carbonate producing organisms globally, resulting in reef degradation and diminished reef growth (e.g.,^73,74^). A decrease in reef framework height and structural complexity significantly increases wave heights above reefs, which will be further exacerbated by rising sea levels^26^. Resulting changes in wave characteristics and cross-reef sediment transport, paired with fluctuations in sediment volume and composition, can dramatically decrease sediment transport potential to islands and shorelines^75^. These ecological and hydrodynamic trends suggest lower net sediment delivery to reef islands under future environmental change, leading to coastal erosion and inundation. Conversely, the legacy of *Halimeda* proliferation as well as increased parrotfish biomass after bleaching suggests that future declines in reef framework production may be partially offset by increases in sediment production^66,67^. This could provide at least some functional compensation as long as the emergent sediment is of an appropriate type and grain size to support sediment landform development^22^. Differences among the studied atolls and islands concerning coral cover, disturbance histories, recovery trajectories and seabird nutrient influence provide a useful set of natural experiments for conceptualising alternative future carbonate budgets. Future studies should consider how shifts in the balance between framework and sediment production—rather than changes in either component alone—may determine the capacity of reef–island systems to keep pace with escalating climatic and anthropogenic pressures.

## Supplementary Information

Below is the link to the electronic supplementary material.

Supplementary Material 1

## Data Availability

All data can be downloaded from the University of Exeter Open Research Repository at https://doi.org/10.24378/exe.32075220. Data files and R code for analysis can also be found at https://github.com/InesLange/carbonate_budgets_chagos/.
