# Supplementary material for: Carbonate framework and sediment production across island-fringing coral reef habitats and a natural nutrient gradient
Source: Sci Rep. 2026 Apr 24;16:18938. doi: 10.1038/s41598-026-49702-w (PMC13275797; doi:10.1038/s41598-026-49702-w)
Supplement: Supplementary file 1 — Supplementary Material 1 [file 41598_2026_49702_MOESM1_ESM.pdf]

Supplementary Material for:

Carbonate framework and sediment production across island-fringing coral reef habitats and a natural nutrient gradient

Ines D Lange, Marleen Stuhr, Chris T Perry, Aitana Gea Neuhaus

Scientific Reports (2026)

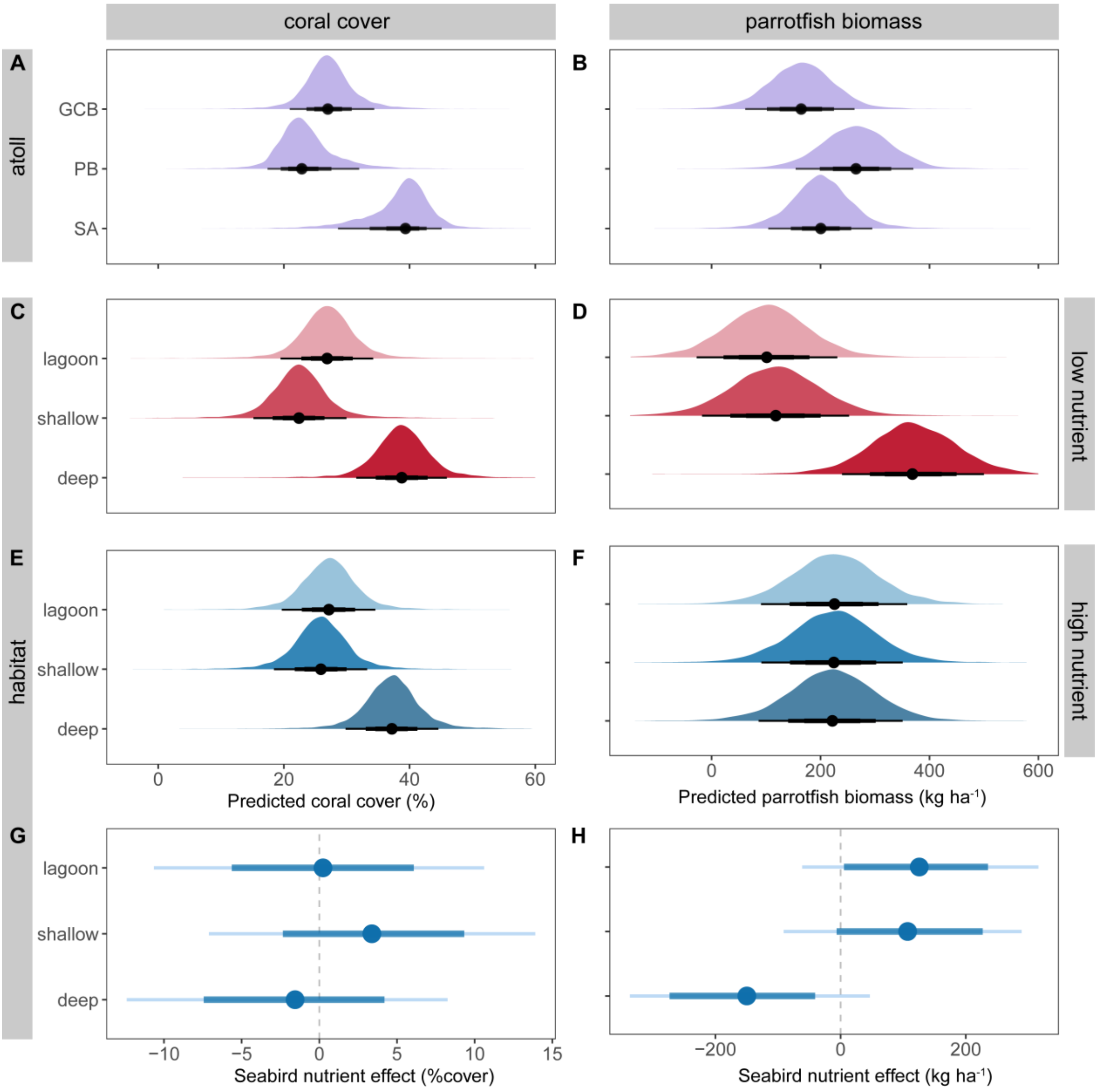

Figure S1: Effects of atoll, habitat and nutrient status on coral cover and parrotfish biomass. Posterior distributions for (A,C,E) coral cover and (B,D,F) parrotfish biomass, comparing (A,B) atolls (Great Chagos Bank, Peros Banhos, Salomon) and (C-F) habitats (lagoon reef, shallow forereef, deep forereef) at low nutrient (C,D) and high nutrient (E,F) sites (linked to seabird densities on islands). Conditional effect of seabird nutrient input on (G) coral cover and (H) parrotfish biomass, with points right of the dashed line indicating a positive effect of nutrients. Points represent median estimates, and lines represent 90% and 70% highest posterior density intervals (HPDIs).

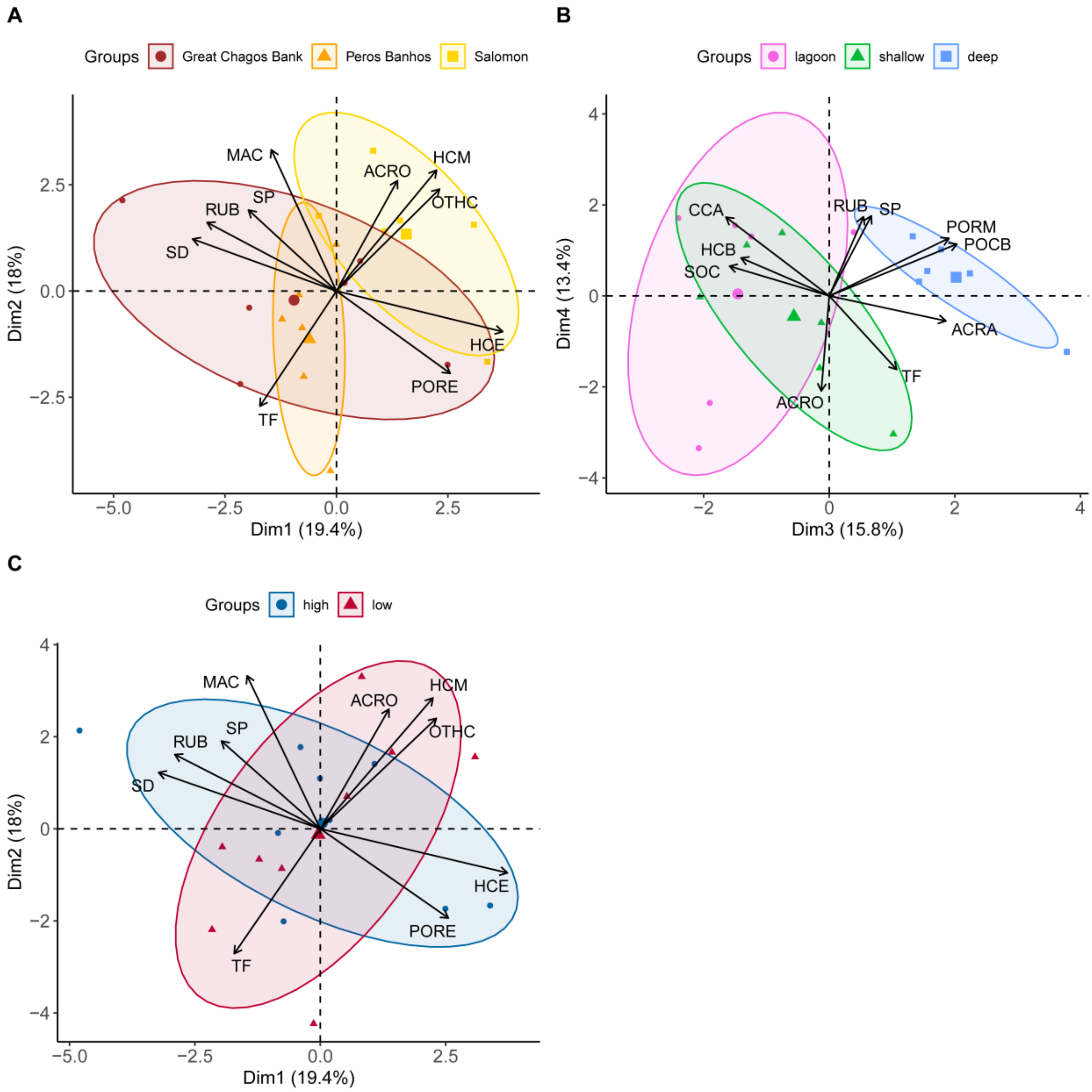

Figure S2: Benthic community composition. (Dis)similarities in benthic community composition between sites projected onto a two-dimensional space using principal component analysis (PCA). Vectors represent benthic covers that are most influential for displayed differences between sites (n = 10) and sites are coloured to compare (A) atolls (PERMANOVA: $F_{2,15} = 3.165$, p = 0.001), (B) habitats (PERMANOVA: $F_{2,15} = 2.638$, p = 0.007) and (C) nutrient status (linked to seabird densities on islands) (PERMANOVA: ns). Ellipse level = 0.7. Abbreviations: ACRA-*Acropora* arborescent, ACRO-*Acropora* corymbose, ACRT-*Acropora* table, POCB-*Pocillopora* branching, HCB-Other branching, PORM-*Porites* massive, HCM-Other (sub)massive hard coral, PORE-*Porites* encrusting, HCE-Other encrusting, FUN-*Fungia* and other solitary taxa, OTHC-foliose/columnar coral morphotypes, CCA-crustose coralline algae, TF-turf algae, MAC-macroalgae, SP-sponges, SOC-soft corals, RUB-rubble, SD-sand.

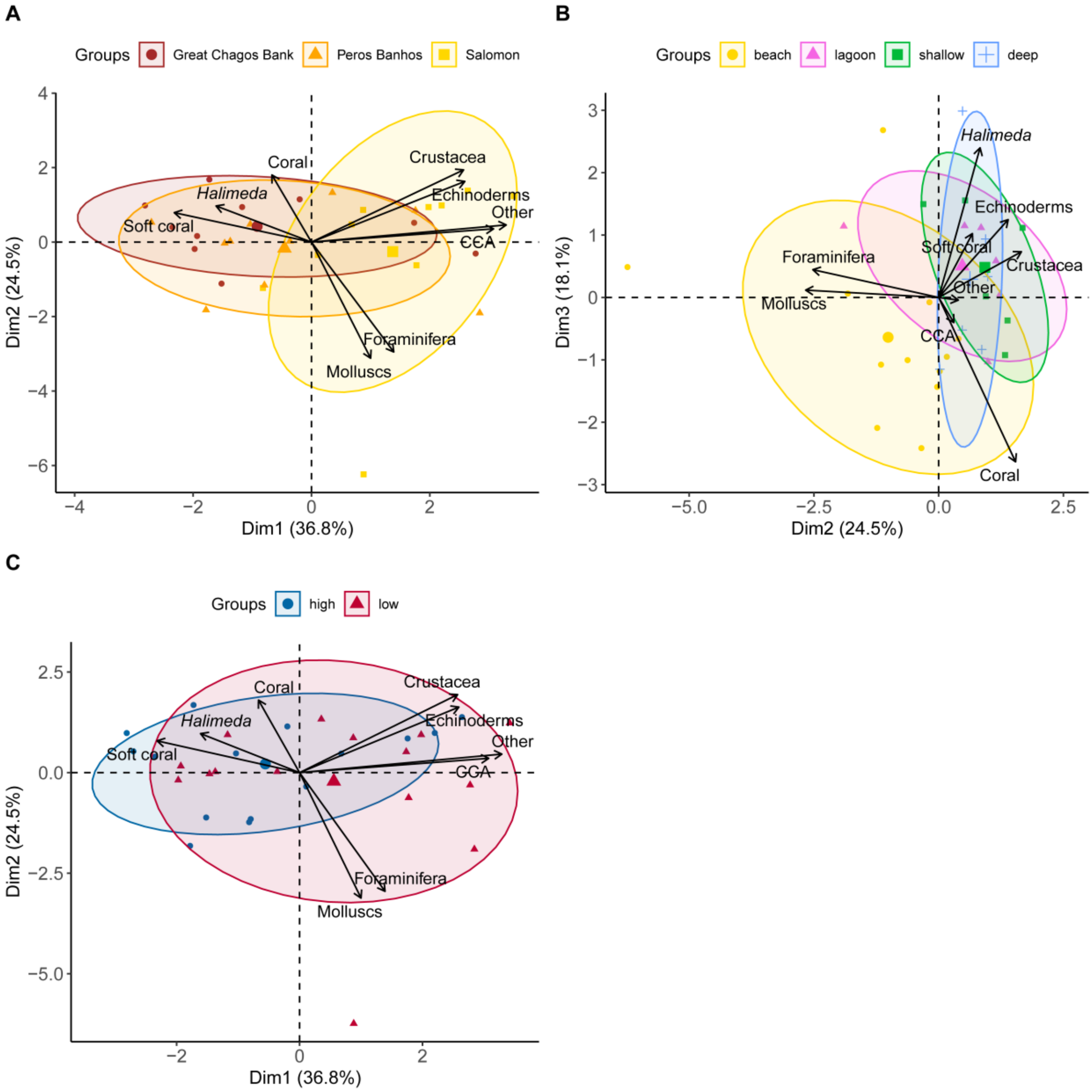

Figure S3: Sediment composition. (Dis)similarities in sediment composition between sites projected onto a two-dimensional space using principal component analysis (PCA). Vectors represent all sediment constituents influencing displayed differences between sites (n = 9), and sites are coloured to compare (A) atolls (PERMANOVA: $F_{2,27} = 3.3001$, p = 0.005), (B) habitats (PERMANOVA: ns) and (C) nutrient status (linked to seabird densities on islands) (PERMANOVA: ns). Ellipse level = 0.7. Abbreviations: CCA-crustose coralline algae.

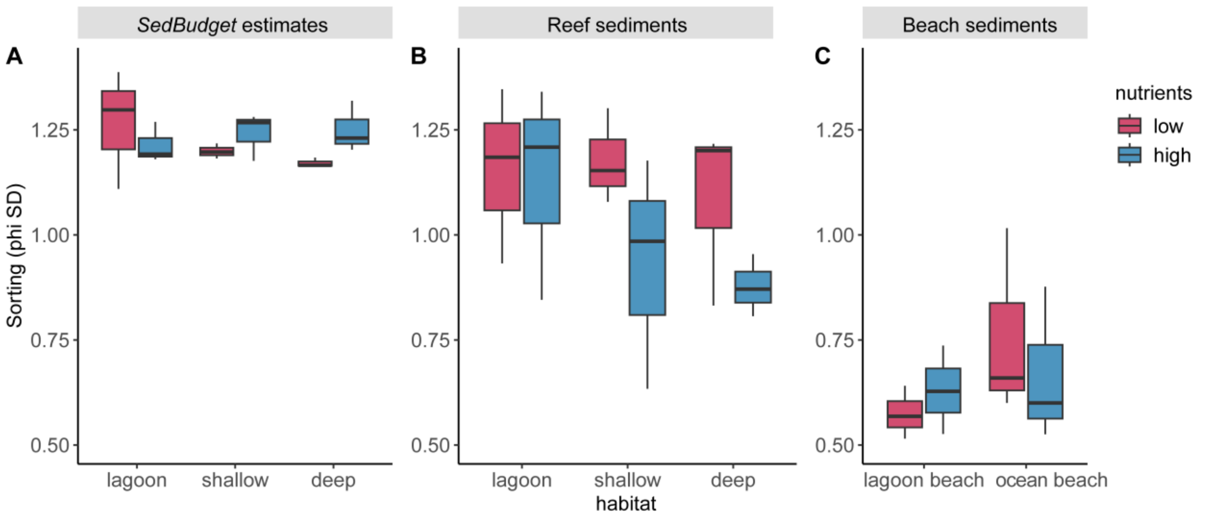

Figure S4: Sorting of sediments. Sorting of sediment samples (phi, geometric standard deviation (SD)) compared across reef habitats (lagoon reef, shallow forereef, deep forereef) and nutrient status (low, high; linked to seabird densities on islands) for (A) *SedBudget* estimates, (B) reef sediments and (C) beach sediments. Sorting was calculated following Folk and Ward (1957), where phi = $-\log_2$(diameter in µm).
